# Efficacy and Safety of Granulocyte-Colony Stimulating Factor Therapy in Chagas Cardiomyopathy: A Phase II Double-Blind, Randomized, Placebo-Controlled Clinical Trial

**DOI:** 10.3389/fcvm.2022.864837

**Published:** 2022-06-09

**Authors:** Carolina T. Macedo, Ticiana F. Larocca, Márcia Noya-Rabelo, Roque Aras, Cristiano R. B. Macedo, Moisés I. Moreira, Alessandra C. Caldas, Jorge A. Torreão, Victor M. A. Monsão, Clarissa L. M. Souza, Juliana F. Vasconcelos, Milena R. Bezerra, Daniela P. Petri, Bruno S. F. Souza, Antônio G. F. Pacheco, André Daher, Ricardo Ribeiro-dos-Santos, Milena B. P. Soares

**Affiliations:** ^1^Department of Cardiology, Hospital São Rafael, Salvador, Brazil; ^2^Gonçalo Moniz Institute, Oswaldo Cruz Foundation, Salvador, Brazil; ^3^Senai Institute on Innovation in Advanced Health Systems, SENAI CIMATEC, Salvador, Brazil; ^4^Escola Bahiana de Medicina e Saúde Pública, Salvador, Brazil; ^5^University Hospital Professor Edgard Santos, Federal University of Bahia, Salvador, Brazil; ^6^Hospital Geral Roberto Santos, Salvador, Brazil; ^7^Center for Biotechnology and Cell Therapy, Hospital São Rafael, Salvador, Brazil; ^8^D’Or Institute for Research and Education, Rio de Janeiro, Brazil; ^9^Scientific Computing Program, Oswaldo Cruz Foundation, Rio de Janeiro, Brazil; ^10^Vice-Presidency of Research and Reference Laboratories, Oswaldo Cruz Foundation, Rio de Janeiro, Brazil

**Keywords:** G-CSF therapy, safety study, cardiac functional analysis, NYHA functional class, Chagas cardiomyopathy

## Abstract

**Aim:**

Previous studies showed that granulocyte-colony stimulating factor (G-CSF) improved heart function in a mice model of Chronic Chagas Cardiomyopathy (CCC). Herein, we report the interim results of the safety and efficacy of G-CSF therapy vs. placebo in adults with Chagas cardiomyopathy.

**Methods:**

Patients with CCC, New York Heart Association (NYHA) functional class II to IV and left ventricular ejection fraction (LVEF) 50% or below were included. A randomization list using blocks of 2 and 4 and an allocation rate of 1:1 was generated by R software which was stratified by functional class. Double blinding was done to both arms and assessors were masked to allocations. All patients received standard heart failure treatment for 2 months before 1:1 randomization to either the G-CSF (10 mcg/kg/day subcutaneously) or placebo group (1 mL of 0.9% saline subcutaneously). The primary endpoint was either maintenance or improvement of NYHA class from baseline to 6–12 months after treatment, and intention-to-treat analysis was used.

**Results:**

We screened 535 patients with CCC in Salvador, Brazil, of whom 37 were randomized. Overall, baseline characteristics were well-balanced between groups. Most patients had NYHA class II heart failure (86.4%); low mean LVEF was 32 ± 7% in the G-CSF group and 33 ± 10% in the placebo group. Frequency of primary endpoint was 78% (95% CI 0.60–0.97) vs. 66% (95% CI 0.40–0.86), *p* = 0.47, at 6 months and 68% (95% CI 0.43–0.87) vs. 72% (95% CI 0.46–0.90), *p* = 0.80, at 12 months in placebo and G-CSF groups, respectively. G-CSF treatment was safe, without any related serious adverse events. There was no difference in mortality between both arms, with five deaths (18.5%) in treatment vs. four (12.5%) in the placebo arm. Exploratory analysis demonstrated that the maximum rate of oxygen consumption during exercise (VO_2_ max) showed an improving trend in the G-CSF group.

**Conclusion:**

G-CSF therapy was safe and well-tolerated in 12 months of follow-up. Although prevention of symptom progression could not be demonstrated in the present study, our results support further investigation of G-CSF therapy in Chagas cardiomyopathy patients.

**Clinical Trial Registration:**

[www.ClinicalTrials.gov], identifier [NCT02154269].

## Introduction

Chronic Chagas cardiomyopathy (CCC) is a life-threatening clinical condition that is responsible for most of the morbidity and mortality caused by infection by the protozoan *Trypanosoma cruzi*. About 30% of asymptomatic infected patients, i.e., those who have the indeterminate form of Chagas disease, will develop cardiac manifestations at some point in their lives, although this might take up to 30 years to occur following the primary infection. The main clinical features include heart failure, sudden death, arrhythmias, stroke, and systemic embolism, with heart failure being the most common cause of death (1–3).

Chronic Chagas cardiomyopathy is the leading cause of non-ischemic heart failure in Latin America, with most of the 2 million cases and 12,500 deaths per year occurring in Brazil and Argentina (4, 5). Chagas disease prevalence is also increasing in Europe and in the United States. In the United States, it is currently estimated that 30,000 to 40,000 individuals may have CCC, and Chagas disease has already been listed by the Centers for Disease Control (CDC) as one of the five most neglected parasitic infections (6).

Although the first reports of Chagas disease date back more than 100 years, the pathogenesis of CCC is still not fully understood. Several studies have demonstrated that the persistence of *T. cruzi* causes permanent myocardial inflammation due to both direct parasite–target response and infection-trigged auto-reactivity, leading to interstitial edema, fibrosis, apoptosis, and chronic myocarditis (7).

Currently, nifurtimox and benznidazole are the only effective anti-trypanosomal therapies available for Chagas disease, but their use is not recommended in patients with chronic cardiomyopathy due to the lack of evidence that these drugs can potentially avert progression of the disease (8, 9). Despite its unique pathophysiological mechanism, standard therapy for CCC is the same as that used to treat any other heart failure syndrome, usually including beta blockers, diuretics, angiotensin-converting enzyme inhibitors (or angiotensin receptor blockers), and spironolactone. Management of late-phase chronic cardiac disease may also include implanted cardiac defibrillators and pacemakers. Although the outcome following heart transplantation in patients with end-stage Chagas cardiomyopathy is better than that observed in patients with non-Chagas disease (10), this procedure is not widely performed in Latin America due to its high costs, limited number of donors, and potential complications (5, 8). Therefore, new therapeutic approaches, mainly targeting specific features of the pathogenic mechanisms of CCC, are urgently needed.

Granulocyte colony-stimulating factor (G-CSF) is a pleotropic cytokine widely used in clinical practice essentially as an adjunctive drug to chemotherapy, due to its ability to induce granulopoiesis and mobilize bone marrow-derived stem cells to the peripheral blood for bone marrow transplantation. The G-CSF receptor is expressed in several cell types, including immune cells, and G-CSF treatment was shown to induce T cell tolerance in several immune-mediated disease models (9). Moreover, there is evidence that G-CSF induces cardiomyogenesis and plays an important role in heart development during embryogenesis (11–13). Therefore, this cytokine has been explored over the last few years as a potential adjuvant therapy for cardiac diseases (14).

In previous studies, it was shown that G-CSF administration improved exercise capacity; reduced inflammation, fibrosis, and tissue parasitism; and modulated the production of pro-inflammatory mediators in a mouse model of CCC (15, 16). These effects were attributed, at least partially, to the immunomodulatory activity of G-CSF (16). In addition, genetically modified mesenchymal stem cells overexpressing G-CSF have also demonstrated immunomodulatory effects by reducing inflammatory mediators, leukocyte infiltration, and myocardial fibrosis (17).

In an attempt to address the lack of effective therapy for CCC, we performed a double-blind, randomized, placebo-controlled clinical trial to evaluate the efficacy and safety of G-CSF therapy with concomitant use of standard heart failure therapy in patients with CCC.

## Methods

### Ethical Approval

The clinical study protocol and informed consent form were reviewed and approved by the Ethics Committee at HSR (Certificate of Presentation of Ethical Appreciation number 22133513.4.0000.0048), which was accredited by the Brazilian National Council on Ethics in Research (CONEP), Ministry of Health. The study was registered at https://www.clinicaltrials.gov on 06/03/2014 (unique identifier: NCT02154269) and was conducted in accordance with Good Clinical Practice Guidelines (GCP) and Brazilian National Health Council resolution 466/20121. During the study, monitoring visits were conducted carried out to ensure GCP adherence.

Written informed consent was obtained from every subject prior to enrollment. If the study subject was illiterate, an impartial third party witnessed the informed consent process. All subjects were informed of the nature and possible associated risks of the trial and that they were free to withdraw their consent to participate at any time. The investigators and study staff ensured the confidentiality of all records.

### Study Design

This study was a double-blind, randomized, placebo-controlled, prospective, comparative superiority trial designed to evaluate the efficacy and safety of G-CSF therapy with concomitant use of standard heart failure therapy in patients with CCC, conducted at two tertiary hospitals in Salvador, northeast Brazil: Hospital São Rafael (HSR) and Hospital Edgard Santos. Double-blinding was limited to the G-CSF and placebo arms.

All patients received standard therapy for heart failure and were randomly assigned to additionally receive either: (a) 10 mcg/kg/day of G-CSF, or (b) 1 mL of 0.9% saline as placebo. Both groups received four cycles of subcutaneous injections for five consecutive days of either saline or G-CSF solution prepared for administration with indistinguishable 1 mL syringes. There were 9-day intervals (a weekend followed by a full week) between cycles. G-CSF was manufactured by the Aché laboratory. If G-CSF therapy effectiveness were demonstrated at the end of the study, patients in the placebo arm were offered G-CSF treatment.

### Study Participants

Between September 24, 2015 and July 30, 2018, 535 patients with Chagas cardiomyopathy were screened in Salvador, Bahia, Brazil, of whom 37 were randomized. The inclusion criteria were previous diagnosis of heart failure as per the Framingham criteria; Chagas disease diagnosis confirmed by two serological tests using distinct methods; age between 20 and 75 years; New York Heart Association (NYHA) functional class II to IV heart failure; and echocardiogram showing left ventricular ejection fraction (LVEF) of 50% or below using the Simpson method. Exclusion criteria were: severe valvular heart disease (except for functional mitral or tricuspid regurgitation); myocardial infarction or history of confirmed coronary artery disease; viral myocarditis; alcohol or drug abuse; acute or chronic kidney disease or previous dialysis therapy; evidence of acute systemic infection; chronic obstructive pulmonary disease with continuous use of steroids or bronchodilators; liver, blood, and neoplastic diseases or hemostasis disorders; chronic inflammatory or infectious diseases; other diseases that could affect life expectancy; any other comorbidity affecting 2-year survival; pregnancy confirmed by human chorionic gonadotropin (β-hCG) testing; and breastfeeding.

### Randomization and Masking

A randomization list using blocks of 2 and 4 and an allocation rate of 1:1 was generated by R software version 3.2. using the Mersenne–Twister method, which was stratified by functional class. The study coordinators provided sealed opaque envelopes. Subjects, clinical study staff, statisticians, and investigators were masked to treatment assignment. Two nurses, a medical investigator, and a hematologist were not blinded. The hematologist was responsible for checking the full blood count on a regular basis in order to monitor G-CSF safety. The blinded investigators did not have access to full blood count tests. The nurses were responsible for preparing and administering the treatments. All laboratory tests, clinical assessments, exams, and imaging were masked to group allocation.

### Procedures

Before randomization, all patients received standard heart failure treatment for 2 months in order to optimize baseline drugs and doses for the follow-up visits. According to the current heart failure guidelines (16), when the study was started, the standard drug regimen for CCC was spironolactone (25 mg/day); furosemide and digoxin, in selected cases; captopril (37.5–150 mg/day); or hydralazine (75 mg/day) with isosorbide mononitrate (20 mg/day), in case of contraindication with angiotensin-converting enzyme inhibitors or angiotensin receptor blockers; carvedilol (6.25–50 mg/day); and amiodarone (200–400 mg/day), if appropriate.

After this 60 days period, all patients underwent clinical evaluation and were submitted to the following baseline tests: urinalysis, blood indices (including full blood count, renal function, electrolytes, liver function, troponin I, ultrasensitive PCR, N-terminal brain natriuretic peptide and cytokines), transthoracic echocardiogram, 6 min walk test, treadmill test, cardiac magnetic resonance, 24 h electrocardiogram (ECG) reading, 12-lead conventional electrocardiogram, urinary and serum β-hCG test (before every treatment cycle, if appropriate), and chest x-ray. The echocardiogram was performed according to current guidelines and the Simpson rule was used to calculate LVEF. Complete follow-up assessments were performed at 6 and 12 months after treatment. Although only two cardiologists followed up the patients clinically, each patient was assessed only once per visit. Regarding the echocardiogram measurements, three cardiologists performed the tests throughout the study, but also each measurement was taken only once per visit as well. Hematologic assessments were done after every treatment cycle.

### Outcomes

The primary outcome, NYHA functional class, was assessed at baseline and at 6, 9, and 12 months of follow-up by a blinded cardiologist. According to the protocol, improvement was defined as either regression or maintenance of baseline functional class within 12 months of follow-up for patients in NYHA class II or III and regression of functional class, or no more than one hospitalization due to acutely decompensated heart failure during follow-up for patients in NYHA class IV.

**FIGURE 1:**
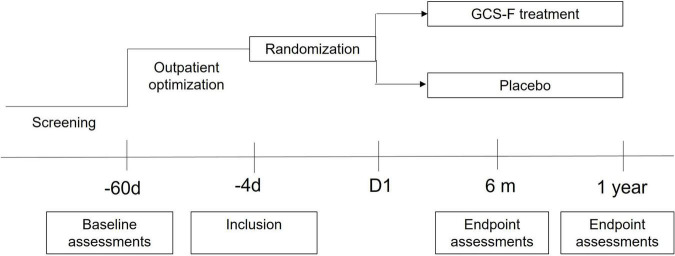
Study time chart. Patients were screened for eligibility before optimization of pharmacological therapy for at least 8 weeks. Patients included in the study were randomized to receive granulocyte-colony stimulating factor (G-CSF) or placebo and were followed up for 12 months.

Secondary efficacy endpoints included LVEF (measured by echocardiogram and cardiac magnetic resonance), functional capacity (assessed by treadmill test and 6-min walking test), quality of life (Minnesota Living with Heart Failure Questionnaire), presence of tachyarrhythmia on 24 h ECG reading, prognostic biomarkers of heart failure (NT-proBNP), and cytokine profile [tumor necrosis alpha (TNF-α) concentration]. The study time chart is presented in Figure 1.

A safety analysis was conducted in the intention-to-treat population, describing frequency, causality, and severity of adverse events (AEs) in each arm. All AEs were subdivided into serious and non-serious events, and were also classified as not related, probably related, or highly likely related to the assigned treatment.

Patients were encouraged to seek immediate advice from the investigators if any AE was suspected. AEs were reported using the Medical Dictionary for Regulatory Activities (MedDra). All clinical or laboratory abnormalities were categorized as grade I to IV according to the Common Terminology Criteria for Adverse Events (CTCAE) of the National Cancer Institute. Any suspected serious AEs (by standard definitions) were reported to the sponsor and to the Ethical Review Committee.

A Data and Safety Monitoring Board (DSMB) was established by the study coordinators, composed of Chagas disease specialists, a cardiologist, a statistician, and another health professional. Meetings occurred periodically, and all changes in the study protocol had to be approved by an independent DSMB and the local ethical committee.

### Statistical Analysis

Study data were collected and managed using Research Electronic Data Capture (REDCap) electronic data capture tools hosted at the Oswaldo Cruz Foundation (FIOCRUZ). REDCap is a secure, web-based software platform designed to support data capture, including double entry.

This study was powered to provide evidence of the superiority of G-CSF treatment over placebo. Based on the current literature, subjects with NYHA functional class II to IV heart failure treated only with standard therapy were expected to have a functional class improvement of 10% in the placebo group and 40% in the G-CSF group during the 12-month follow-up. Thus, for a 30% absolute difference in NYHA class to be detected with a 95% confidence level and 80% statistical power, a sample size of 29 subjects per treatment group was needed. Taking into account a 15% loss of follow-up in 12 months, the final sample size was 35 subjects per treatment group. An interim analysis with half the sample size was planned in the DSMB charter. Those interim results are presented here, using intention-to-treat analysis.

Primary endpoint analysis was done by intention-to-treat and for missing data, last observation carrying forward as replacement strategy was used, and death before the final assessment was considered as treatment failure.

Baseline characteristics and adverse events of the study population were summarized using descriptive statistics with frequencies and percentages for categorical variables. For numerical variables, mean and standard deviation (SD) values were used.

The proportions of categorical variables were compared using Pearson’s chi-squared test with Yates’s continuity correction at a significance level of 5%. Differences in continuous variables between groups were compared using the *t*-test or Mann–Whitney test. Two-sided *p*-values < 0.05 were considered statistically significant. Analyses were performed using IBM SPSS statistics software version 25.

## Results

**FIGURE 2:**
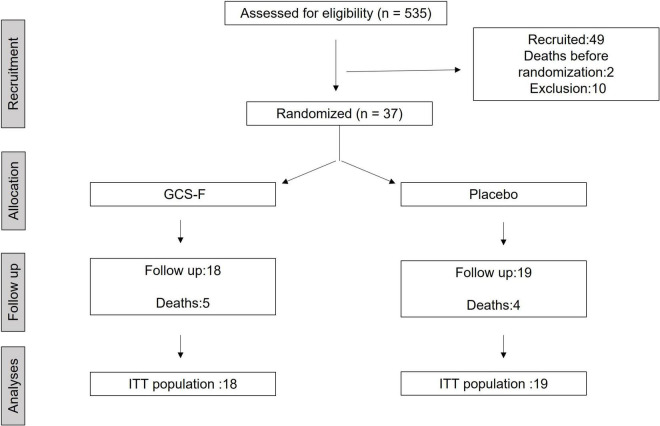
Flowchart showing the flow of patients throughout the trial. ITT, intention-to-treat.

A total of 535 subjects were assessed for eligibility at both trial sites (Hospital São Rafael and Hospital Edgard Santos) in Salvador, Brazil, from September 2015 to October 2018. Thirty-seven subjects were randomized (1:1) to one of the treatment arms: (a) G-CSF (10 mcg/kg/day) with concomitant use of standard heart failure therapy, or (b) 0.9% saline plus standard heart failure therapy (placebo). Eighteen subjects were allocated to the treatment group and 19 to the placebo group. The main reason for not including most subjects was unavailability for follow-up, as the vast majority of patients lived in the countryside or remote areas. Nine patients died during follow-up (Figure 2).

**FIGURE 3:**
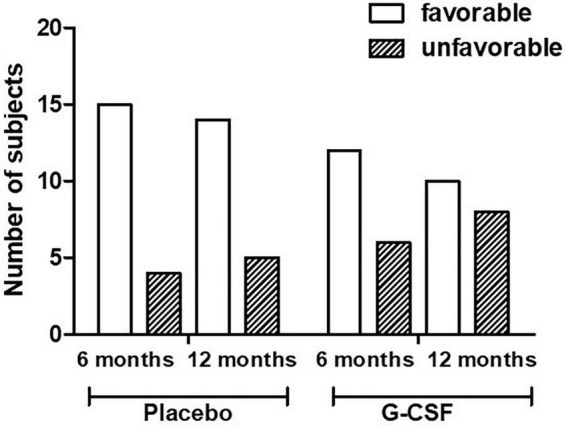
Progression of NYHA functional class at 6 and 12 months (*p* = 0.47 and 0.80, respectively). Favorable: Improvement of NYHA class; Unfavorable: Worsening of NYHA class.

**FIGURE 4:**
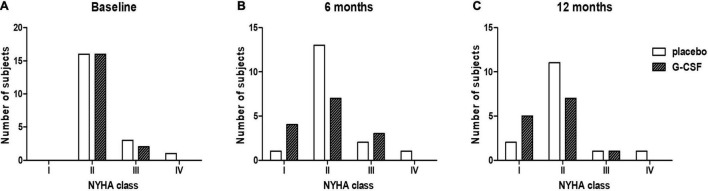
NYHA class distributions at baseline (T = 0) **(A)**, at 6 months **(B)** and 12 months **(C)** after treatment (*p* = 0.20 and 0.38, respectively).

The baseline characteristics of the subjects were similar between the treatment groups (Table 1). The primary outcome was measured by evaluating NYHA functional class improvement, comparing baseline with 6 and 12-month data. All 37 subjects were included in this intent-to-treat analysis. Death before end of follow-up was considered as deterioration. The overall frequency of either maintenance or improvement of clinical condition was primary endpoint was 78% (95% CI 0.60–0.97) vs. 66% (95% CI 0.40–0.86), *p* = 0.47, at 6 months and 68% (95% CI 0.43–0.87) vs. 72% (95% CI 0.46–0.90), *p* = 0.80, at 12 months in placebo and G-CSF groups, respectively (Figure 3). The distribution across the NYHA classes in both groups at inclusion (*T* = 0) and at 6 and 12 months after treatment is shown in Figure 4.

**TABLE 1:** Baseline characteristics of subjects.

		Treatment groups	
		Placebo + SHFT (19 subjects)	G-CSF + SHFT (18 subjects)
Gender	Male	11 (57.8%)	10 (55.0%)
NYHA class	II	16 (43.2%)	16 (43.2%)
	III or IV	3 (8.1%)	2 (5.4%)
Hypertension	No	12 (63%)	14 (77%)
Dyslipidemia	No	12 (63%)	9 (50%)
Age (years)		59 (±8)	60 (±8)
BMI		24.38 (±7.14)	23.33 (±5.94)
LVEF (Simpson method)		33 (±10)	32 (±7)

*Absolute number and percentage (%) were used to present categorical variables. Quantitative data presented using mean ± standard deviation (SD). SHFT, standard heart failure therapy; NYHA, New York Heart Association; BMI, body mass index; LVEF, left ventricular ejection fraction.*

The secondary outcomes included improvement of LVEF (as measured by echocardiogram) at 6 and 12 months compared to baseline results (Table 2). The frequency of improvement in placebo and G-CSF arms was 23 vs. 26% (*p* = 0.83) at 6 months and 31 vs. 20% at 12 months, respectively (*p* = 0.47). The difference in percentage of myocardial fibrosis measured by cardiac magnetic resonance at baseline and 12 months between placebo (23.5%) and G-CSF (30.6%) groups was not significant (*p* = 0.23).

**TABLE 2:** Secondary endpoints at baseline and 12 months after treatment.

	Group	Baseline	*p*-value	12 months	*p*-value
LVEF (%)	Placebo	33 (±10.7)	0.63	35 (±12)	0.24
	G-CSF	31 (±7.8)		31 (±6.7)	
VO_2_ max	Placebo	22.7 (±10.7)	0.95	20 (±12)	0.064
	G-CSF	22.9 (±7.8)		26 (±6.7)	
Myocardial fibrosis (%)	Placebo	23.8 (±13.4)	0.75	23.5 (±13.7)	0.23
	G-CSF	25.5 (±14)		30.6 (±9.9)	
Six minutes walking test	Placebo	450 (390–480)	0.86	450 (348–502)	1.0
	G-CSF	456 (390–471)		429 (360–495)	
MLHLQ	Placebo	43.2 (±19.5)	0.43	35.1 (±17.3)	0.23
	G-CSF	38.4 (±16.7)		26 (±20.5)	

*LVEF, left ventricular ejection fraction; VO_2_ max, maximum oxygen consumption during exercise (mL/kg/min). Myocardial fibrosis evaluated by cardiac magnetic resonance. Six-minute walking test distance: median (first to third quartile). Scores for Minnesota Living with Heart Failure (MLHFQ) physical subscale range from 0 to 40, emotional subscale from 0 to 25, and total scale from 0 to 105, with higher scores indicating worse health status.*

Twenty-two subjects performed a treadmill test at enrollment and the last follow-up visit. Nine subjects (41%) had 10% higher oxygen consumption than at baseline. Although not statistically significant, the maximum oxygen consumption at 12 months was better in the G-CSF group than in the placebo group, showing a tendency of improvement (*p* = 0.06). Quality of life was assessed using the Minnesota questionnaire at randomization and at 6 and 12 months. An independent samples median test of the total score showed no statistical difference between the groups at 6 or 12 months (*p* = 1.0 and 0.69).

Analysis of incidence of non-sustained ventricular tachycardia (NSVT) was performed by 24 h ECG monitoring. At 12 months, the median NSVT was maintained in the G-CSF group and increased in the placebo group compared to baseline, even though no statistical significance was found (*p* = 0.7).

N-terminal pro-BNP (NT-proBNP) concentration was measured at enrollment and at 12 months, and no statistical significance was detected between the groups at both time points (*p* = 0.47 and 0.86, respectively) (Supplementary Table 1). Finally, tumor necrosis factor-alpha (TNF-α) concentration was measured as an exploratory analysis. We found a significant increase in TNF-α concentration at days 5 and 47 during the G-CSF application period, followed by a decrease in concentration until the values were similar to those in the placebo group at 12 months (*p* = 0.69) (Supplementary Table 2).

There were 11 deaths during the study, two of which happened before randomization. Overall mortality was 24.3% and, out of the nine remaining deaths, five occurred in the treatment group and four in the placebo group. Fifty-nine serious adverse events (SAE) were reported, none of which were related to G-CSF treatment. The classification of SAEs and their frequency per treatment group is presented in Table 3.

**TABLE 3:** Classification and frequency of serious adverse events (SAEs) per treatment group.

SAE	Placebo (32 SAEs) *n* (%)	G-CSF (27 SAEs) *n* (%)
Death	4 (12.5%)	5 (18.5%)
Life-threatening	8 (25.0%)	7 (25.9%)
Inpatient hospitalization or prolonged existing hospitalization	20 (62.5%)	9 (33.3%)
Clinically significant	0 (0%)	6 (22.2%)

*n: total number of events in each group, not number of patients who had events.*

A total of 283 adverse events were reported using MedDra. The AEs were aggregated using the system organ classes (SOC), and those with a frequency higher than 3% in either treatment arm are presented in Table 4.

**TABLE 4:** AEs with a frequency higher than 3.0% per treatment arm.

AE	Placebo (108 AEs) *n* (%)	G-CSF (148 AEs) *n* (%)
Cardiac disorders	7 (6.4%)	5 (3.3%)
Gastrointestinal disorders	28 (25.9%)	25 (16.8%)
General disorders and administration site conditions	16 (14.8%)	31 (20.9%)
Infections and infestations	5 (4.6%)	14 (9.4%)
Investigations (other clinical disorders investigated)*	4 (3.7%)	5 (3.3%)
Metabolic and nutrition disorders	6 (5.5%)	1 (0.6%)
Musculoskeletal and connective tissue disorders	13 (12.0%)	18 (12.1%)
Nervous system disorders	12 (11.1%)	16 (10.8%)
Reproductive system and breast disorders	1 (0.9%)	6 (4.0%)
Respiratory, thoracic, and mediastinal disorders	11 (10.1%)	19 (12.8%)
Skin and subcutaneous tissue disorders	1 (0.9%)	6 (4.0%)
Vascular disorders	4 (3.7%)	2 (1.3%)

*Total number of events in each group, not number of patients who had events.

## Discussion

Chronic Chagas cardiomyopathy is a life-threatening condition for which no specific and effective treatment has been found. Since drug repurposing is the quickest research and development strategy to deliver a new treatment, our group designed this trial in an attempt to repurpose G-CSF as a potential treatment for CCC, given its well-established safety profile and promising results in experimental models of this disease. The present study was the first randomized clinical trial assessing the effects of therapy with G-CSF in symptomatic patients with CCC. Although it was well-tolerated, no statistically significant clinical improvement was demonstrated in terms of efficacy.

The New York Heart Failure (NYHF) classification has been shown to be an independent predictor of mortality in patients with chronic heart failure, including Chagas cardiomyopathy (18). Additionally, it is easily applied, well-validated, and widely used for risk stratification in heart failure. In this study, for most of the subjects, their NYHA classification was either maintained or improved during the study follow-up. This may have been the result of standard heart failure treatment optimization before randomization and close monitoring throughout the follow-up, as the same trend was observed in both study arms. No parameters used to evaluate the influence of G-CSF treatment were statistically significant, including the primary outcome (NYHA); however, there was a trend showing that G-CSF treatment could potentially avert the natural progression of CCC, promoting some improvement in the clinical condition of patients 6 months after treatment. This may indicate that a different treatment regimen, with courses of G-CSF distributed over longer periods, might be more effective in inducing prolonged improvement in CCC.

Regarding cardiomyopathies with other etiologies and the use of G-CSF, the overall mortality in our study was higher (24.3%). A study recently published on ischemic cardiomyopathy showed an overall mortality of 15.8% with no statistical difference between G-CSF and placebo groups (19). However, we did not expect to have a similar mortality rate, as it is well-known that CCC carries a worse prognosis with a higher mortality rate compared to heart failure with other etiologies. In a recent study, mortality from cardiovascular and other causes was 40% higher in patients with CCC than in patients with ischemic heart disease (20). Our study might more accurately reflect this aspect, as we had a follow-up period of 12 months. Our mortality rate is in keeping with what was described by Shen and collaborators. Their overall mortality rate by CCC was 29.2% in a study with more than 2,000 patients comparing cardiovascular mortality and hospitalization between heart diseases with different etiologies, including Chagas (20, 21).

The relationship between myocardial fibrosis with ventricular dysfunction and cardiac arrhythmias in patients with CCC is well-established (22). In our study, assessment of fibrosis by cardiac magnetic resonance showed a trend in regression of myocardial fibrosis in 18.8% of patients, without a significant difference between the groups. This reduction in the percentage of myocardial fibrosis, along with some improvement in the incidence of cardiac arrhythmias, was previously demonstrated by our group in an experimental model of CCC (15, 17). A possible explanation for the better therapeutic response in mice is the earlier time of intervention during the course of the disease, when the inflammatory response and fibrosis may be more effectively modulated by G-CSF.

The influence of the timing of G-CSF administration on its effects has been suggested in the literature. Several studies demonstrated that earlier application of G-CSF led to further improvement in parameters such as functional class, left ventricular function, and cardiac remodeling (19, 23–25). In our study, G-CSF therapy was carried out at an advanced stage of the disease, in patients with greatly reduced left ventricular function. It is reasonable to suppose that earlier administration of G-CSF could potentially promote better results. Additionally, the worst response to G-CSF in patients with Chagas disease, compared to other cardiomyopathies, might also be related to differences in their pathogenesis. In addition to the presence of the parasite, CCC has greater myocardial inflammatory infiltrates, as shown by an analysis of explanted hearts from patients with CCC compared to other etiologies of heart failure (26).

Other studies have also shown that G-CSF can improve left ventricular function and reduce cardiac remodeling and TNF-α levels (16, 19, 24). Here we found a transient increase in G-CSF-treated group, which decreased to levels similar to those of placebo group at the end of follow-up. We also measured the concentration of NT-proBNP, a biomarker that is strongly correlated with NYHA functional class, as previously described (27). No statistically significant differences in NT-proBNP concentration between the experimental groups were demonstrated.

Regarding exercise tolerance, the maximum rate of oxygen consumption during exercise (VO_2_) is an index widely used to objectively assess exercise capacity and cardiovascular reserve. Szlachcic et al. demonstrated that the survival rate in individuals with VO_2_ levels greater than 10 mL/kg/min was 80%, whereas it was 20% in those with lower VO_2_ levels (28). Mady et al. demonstrated that the VO_2_ level, along with LVEF, was an independent predictor of mortality during a 30-month follow-up of patients with CCC (29). In our study, there was a trend of improved VO_2_ in the G-CSF group at 12 months, but it did not reach statistical significance.

For quality-of-life assessment, the Minnesota Living with Heart Failure (MLHF) questionnaire was used. Although not statistically significant, the difference between groups was greater in the final evaluation at 12 months, with a tendency toward lower scores (better quality of life) in the G-CSF group.

The follow-up duration may also have had an influence on the results. Although 12 months might have been adequate for assessing functional class and quality of life, this period is unlikely to be long enough to show differences in parameters with slower and more progressive changes, such as left ventricular function, myocardial fibrosis, and functional capacity on exercise testing. In fact, assessment of G-CSF treatment showed benefits when patients with ischemic cardiomyopathy were evaluated 10 years after treatment (19).

### Potential Limitations

A low recruitment rate, possible measurement bias, and a lack of statistically significant results are the main limitations of our study and were considered relevant enough to halt the study following the interim analysis. Despite its underpower to both efficacy and safety outcomes, our study suggests that the treatment with G-CSF is safe in patients with chronic Chagas disease cardiomyopathy. G-CSF has been widely applied in clinical practice with few serious side effects (25, 30–33).

## Conclusion

This study is the first randomized clinical trial to assess the effects of G-CSF in Chagas cardiomyopathy. Since its efficacy in preventing the clinical progression of the disease could not be demonstrated in the present study, G-CSF therapy shall not be advised as a standard treatment for this condition. Further investigations need to be carried out in order to confirm the safety and evaluate the possible efficacy of the G-CSF in Chagas cardiomyopathy, possibly testing the administration of G-CSF earlier in the course of the disease, and with a longer follow-up period.

## Data Availability Statement

The original contributions presented in the study are included in the article/Supplementary Material, further inquiries can be directed to the corresponding author/s.

## Ethics Statement

The studies involving human participants were reviewed and approved by Ethics Committee at HSR (Certificate of Presentation of Ethical Appreciation number 22133513.4.0000.0048). The patients/participants provided their written informed consent to participate in this study.

## Author Contributions

AD, CaM, TL, MS, RR-d-S, and AP participated in the study concept and design. CaM and TL were the principal investigators. CaM, TL, MB, DP, BS, CS, MN-R, RA, and CrM conducted data collection and quality assurance. JV, VM, MM, AC, and JT performed imaging exams and laboratory analysis. AD, CaM, and AP performed data analysis. AD, CaM, TL, MS, and AP participated in interpretation of the data and writing and critical revision of the manuscript. All authors have read and agreed to the published version of the manuscript.

## Conflict of Interest

The authors declare that the research was conducted in the absence of any commercial or financial relationships that could be construed as a potential conflict of interest.

## Publisher’s Note

All claims expressed in this article are solely those of the authors and do not necessarily represent those of their affiliated organizations, or those of the publisher, the editors and the reviewers. Any product that may be evaluated in this article, or claim that may be made by its manufacturer, is not guaranteed or endorsed by the publisher.

## Abbreviations

CCC: chronic Chagas cardiomyopathy
G-CSF: granulocyte-colony stimulating factor
NYHA, class: New York Heart Association functional class
CDC: centers for disease control
LVEF: left ventricular ejection fraction
NT-proBNP: N-terminal brain natriuretic peptide
ECG: electrocardiogram
TNF- α: tumor necrosis alpha
AE: adverse events
MLHF: Minnesota Living with Heart Failure.
