# Supplementary material for: Efficacy and Safety of Granulocyte-Colony Stimulating Factor Therapy in Chagas Cardiomyopathy: A Phase II Double-Blind, Randomized, Placebo-Controlled Clinical Trial
Source: Front Cardiovasc Med. 2022 Jun 9;9:864837. doi: 10.3389/fcvm.2022.864837 (PMC9222127; doi:10.3389/fcvm.2022.864837)
Supplement: Supplementary file 1 [file Table_1.pdf]

Efficacy and Safety of Granulocyte-Colony Stimulating Factor Therapy in Chagas Cardiomyopathy: A Phase II Double-Blind, Randomized, Placebo-Controlled Clinical Trial

Carolina T. Macedo[1,2,3†], Ticiana F. Larocca[2†], Márcia Noya-Rabelo[1,4], Roque Aras Junior[5], Cristiano R. B. Macedo[5], Moisés I. Moreira[1], Alessandra C. Caldas[1], Jorge A. Torreão[1], Victor M. A. Monsão[6], Clarissa L. M. Souza[5], Juliana F. Vasconcelos[2,4], Milena R. Bezerra[3], Daniela P. Petri[7], Bruno S. F. Souza[2,7,8], Antônio G. F. Pacheco[9], André Daher[10], Ricardo Ribeiro-dos-Santos[2,3], Milena B. P. Soares[2,3]*

[1]Department of Cardiology, Hospital São Rafael, Salvador, Brazil, [2]Gonçalo Moniz Institute, Oswaldo Cruz Foundation (FIOCRUZ), Salvador, Brazil, [3]Senai Institute on Innovation in Advanced Health Systems, SENAI CIMATEC, Salvador, Brazil, [4]Escola Bahiana de Medicina e Saúde Pública, Salvador, Brazil, [5]University Hospital Prof. Edgard Santos, Federal University of Bahia, Salvador, Brazil, [6]Hospital Geral Roberto Santos, Salvador, Brazil, [7]Center for Biotechnology and Cell Therapy, Hospital São Rafael, Salvador, Brazil, [8]D'Or Institute for Research and Education (IDOR), Rio de Janeiro, Brazil, [9]Scientific Computing Program (PROCC), Oswaldo Cruz Foundation (FIOCRUZ), Rio de Janeiro, Brazil, [10]Vice-Presidency of Research and Reference Laboratories, Oswaldo Cruz Foundation (FIOCRUZ), Rio de Janeiro, Brazil.

[*]**Correspondence to:** Prof. Milena B. P. Soares.
milena.soares@fiocruz.br
†These authors have contributed equally to this work.

Supplementary Tables

Table S1. NT-proBNP concentration per treatment group (pg/ml).

	Treatment group	N	Mean	P Value (G-CSF vs Placebo)
**Baseline**	Placebo	19	1574.74 (±1354.19)	0.47
	G-CSF	16	2137.88 (±2798.59)	
**12 months**	Placebo	14	1854.71 (±2269.68)	0.86
	G-CSF	11	1704.73 (±1855.99)	

Values are mean ± standard deviation. NT-BNP= N-terminal pro hormone of brain natriuretic peptide.

Table S2. TNF-α mean concentrations and standard deviation (SD) in pg/ml.

	Treatment group		
	Placebo	G-CSF	P value
	Mean	Mean	G-CSF vs Placebo
**D1**	1.58 (±0.64)	1.51 (±0.63)	0.767
**D5**	1.63 (±0.73)	2.62 (±1.02)	0.010
**D47**	1.34 (±0.48)	2.11 (±0.69)	0.002
**12 months**	1.28 (±0.69)	1.40 (±0.88)	0.692

Values are mean ± standard deviation. D1, D5, D47 and 12 months are the days that blood samples were collected.
